# Intestinal bile acids provide a surmountable barrier against *C. difficile* TcdB-induced disease pathogenesis

**DOI:** 10.1073/pnas.2301252120

**Published:** 2023-05-01

**Authors:** Simoun Icho, Jennifer S. Ward, John Tam, Larry K. Kociolek, Casey M. Theriot, Roman A. Melnyk

**Affiliations:** ^a^Molecular Medicine Program, The Hospital for Sick Children Research Institute, Toronto, ON M5G 0A4; ^b^Department of Biochemistry, University of Toronto, Toronto, ON M5S 1A8; ^c^Ann & Robert H. Lurie, Children’s Hospital of Chicago, Chicago, IL 60611; ^d^Department of Pediatrics, Division of Pediatric Infectious Diseases, Northwestern University Feinberg School of Medicine, Chicago, IL 60611; ^e^Department of Population Health and Pathobiology, College of Veterinary Medicine, North Carolina State University, Raleigh, NC 27606

**Keywords:** Toxin, Host, *C. difficile*, bile acid, pathogenesis

## Abstract

Intestinal bile acids play an essential role in the *Clostridioides difficile* lifecycle having been shown in vitro to modulate various aspects of pathogenesis, including spore germination, vegetative growth, and more recently the action of the primary virulence determinant, TcdB. Here, we investigated whether physiological levels of the total pool of intestinal bile acids in mice and humans protect against TcdB action. Small molecules extracted from the lumenal contents of the small intestine, cecum, colon, and feces were found to inhibit TcdB in accordance with the differential amounts of total bile acids in each compartment. Extracts from antibiotic-treated and germ-free mice, despite harboring dramatically altered bile acid profiles, unexpectedly also prevented TcdB-induced cell rounding to similar extents. We show that protection, however, is surmountable and can be overcome at higher doses of TcdB—typical to those seen during severe *C. difficile* infection—suggesting that the protective properties of intestinal bile acids are operant primarily under low to moderate toxin levels. Taken together, these findings demonstrate a role for intestinal bile acids in attenuating virulence, provide insights into asymptomatic carriage of toxigenic *C. difficile*, and inform strategies to manipulate bile acid levels for therapeutic benefit.

## Significance

*Clostridioides difficile* is the leading cause of hospital-acquired diarrhea worldwide. While TcdB, the primary virulence determinant of *C. difficile*, is known to be required for the intestinal symptoms associated with infection, asymptomatic carriage of TcdB-positive *C. difficile* is common, suggesting that specific host factors may influence disease severity. Here, we show that small molecules extracted from intestinal samples from mice and humans protect against low to moderate levels of TcdB; however, at higher concentrations of toxin, inhibition is overcome. We show that intestinal bile acids, which directly bind and inhibit TcdB, are responsible for the protection against TcdB. These findings demonstrate a role for intestinal bile acids in virulence and provide an anti-toxin approach to treat disease.

*Clostridioides difficile* (formerly *Clostridium difficile*) is a gram-positive, spore-forming bacterium that has emerged as a major public health concern in recent decades. *C. difficile* is transmitted via the fecal-oral route as spores that germinates in response to environmental signals within the gastrointestinal tract (1, 2). In individuals whose protective indigenous microbiota has been perturbed by antibiotics, vegetative *C. difficile* bacteria thrive and colonize the susceptible host (3). Virulent strains of *C. difficile* produce gut-damaging protein toxins that are responsible for the clinical symptoms of disease, which can range from self-limiting diarrhea to pseudomembranous colitis, and potentially death in more severe cases (3). In particular, the homologous toxins TcdA and TcdB produced by pathogenic strains of *C. difficile* are capable of causing disease in animal models (4), with TcdB appearing to be the primary determinant of disease in humans (5). Interestingly, although TcdB is necessary for symptomatic *C. difficile* infection (CDI), it does not appear to be sufficient for disease as asymptomatic carriage of TcdB-positive *C. difficile* is common, especially in hospitals and healthcare settings and in certain populations including newborns (6), suggesting that additional factors and conditions play a role in determining disease symptom severity (7–9). Elucidating the host- and microbiota-derived factors that contribute to *C. difficile* disease is important both for better understanding disease pathogenesis and for rationally designing novel therapeutic strategies to treat CDI.

A family of intestinal metabolites that have been shown to play a central role throughout the *C. difficile* lifecycle are primary and secondary bile acids (10). The primary bile acid taurocholic acid and other cholic acid derivatives have been shown to trigger germination of *C. difficile* spores into their toxin-producing vegetative state via binding to the germinant receptor CspC (1, 11, 12), while chenodeoxycholic acid derivatives and other secondary bile acids inhibit cholic acid-induced germination (12, 13). Moreover, the microbially derived secondary bile acids, including deoxycholic acid and lithocholic acid, inhibit growth of *C. difficile* (14–16). Studies investigating the gut metabolome in mice before and after antibiotic exposure have shown that *C. difficile* can exploit specific metabolites that become more abundant in the mouse gut after antibiotics, such as taurocholic acid for spore germination (16). Bile acids have also been shown to modulate the *C. difficile* lifecycle indirectly through effects on host response (17, 18). Further, *Clostridium scindens*, an intestinal bacterium capable of 7α-dehydroxylating bile acids was shown to enhance resistance to CDI by producing key bile acids that directly inhibit *C. difficile* outgrowth (19). Remarkably, the associations between bile acids and the *C. difficile* lifecycle extend beyond the bacterium, spore, and host. More recently, we found that bile acids also directly interact with TcdB in the combined repetitive oligopeptide domain region and induce a major conformational change in the toxin that prevents its ability to bind and intoxicate host cells (20). This finding uncovered an intersection between bile acids and the *C. difficile* lifecycle and prompted us to speculate that bile acids may play a role in vivo in modulating bacterial virulence and thus disease symptom severity.

In this study, we extracted small-molecule metabolites directly from intestinal and fecal contents of healthy, antibiotic-treated, and germ-free mice and healthy human subjects with the goal of determining whether and to what extent physiological levels of bile acids (and any other metabolites) are able to inhibit cytopathic levels of TcdB. Small-molecule extracts from all groups and from different intestinal compartments dose-dependently inhibited TcdB to varying degrees. Based on this, we undertook a series of studies to demonstrate that bile acids were responsible for the observed effects. Based on our unexpected finding that bile acids are protective in mice that are susceptible to CDI, we explored the interplay between bile acids and toxins and found that the degree of protection afforded by bile acids was tunable and could be manipulated to provide temporal and spatial regulation of *C. difficile* virulence.

## Results

### Small-Molecule Metabolite Extraction from Mouse Intestinal and Stool Contents.

To establish whether in vivo physiologically relevant levels of bile acids inhibit TcdB intoxication, we set out initially to develop a robust method to extract bile acids and other small molecule metabolites from intestinal contents and stool. The goal was to develop a protocol that was stringent enough to exclude or inactivate potentially confounding factors from biological samples, such as living organisms and proteases, without affecting the yields and integrity of the bile acids and other small molecule metabolites in the biological samples. The lumenal contents from the small intestine, cecum, and colon sections of healthy C57BL/6 mice along with fecal pellets were harvested, weighed, and flash frozen. The samples were subsequently thawed and resuspended in serum-free media (SFM) to a final concentration of 1 mg/mL of sample wet weight and then sterile filtered using a 0.22-μm membrane to remove intact organisms and cellular debris. The filtered supernatants were then boiled for 10 min to denature proteins and eliminate any remaining microbes that may have escaped filtration. The processed extracts were both sterile and also free of cytotoxic agents that were effectively removed by the boiling step (*SI Appendix*, Fig. S1).

To evaluate the impact of the extraction protocol on the integrity of bile acids, individual primary and secondary bile acids were spiked into SFM and then the total amounts of each bile acid were quantified after each step of the extraction procedure. Using liquid chromatography-mass spectrometry (LC/MS), we showed that each of the ten bile acids was retained throughout the process, with no apparent loss in concentration (*SI Appendix*, Fig. S2*A*). Further, we tested the ability of each bile acid to inhibit TcdB-induced cell rounding of human lung fibroblasts after 3-h and saw no change in the potency before and after extraction indicating that the extraction procedure neither excludes nor damages the chemical integrity of bile acids (*SI Appendix*, Fig. S2*B*).

### Small Molecules Extracted from Mouse Intestinal and Fecal Contents Inhibit TcdB.

**Fig. 1.**
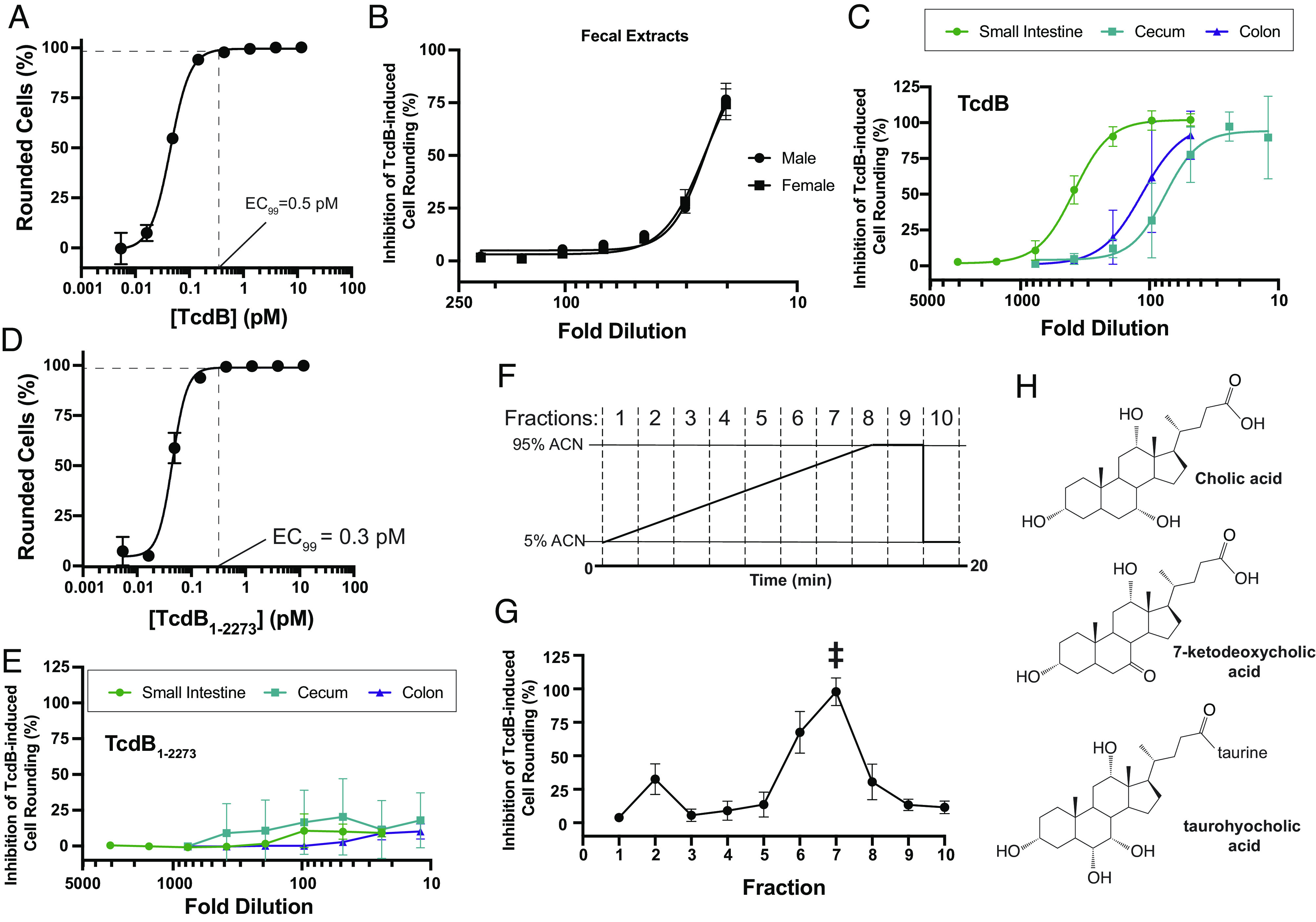
Intestinal bile acids from healthy mice protect against *C. difficile* toxin B (TcdB). (*A*) TcdB-mediated cell rounding of human lung fibroblast IMR-90 cells. Experiment was conducted in triplicate (n = 3) and error bars denote (SEM). (*B*) IMR-90 cells were treated with 0.5 pM TcdB (EC_99_) and increasing dilutions of sterile-filtered and boiled fecal content of male or female C57BL/6 mice. Inhibition of TcdB-mediated cell rounding was calculated as the ratio of the difference between the toxin- and fecal content-treated and healthy cells to toxin-treated and healthy cells. Experiments were conducted in quadruplicate (n = 4) and error bars denote SEM. (*C*) IMR-90 cells were treated with 0.5 pM TcdB (EC_99_) and increasing dilutions of sterile-filtered and boiled small intestinal, cecal, and colon contents. Inhibition of TcdB-mediated cell rounding was calculated as above, experiments were conducted at n = 5 to 6, and error bars denote (SD). (*D*) Truncated and bile-acid insensitive TcdB_1-2273_-mediated cell rounding of IMR-90 cells. Experiment was conducted in triplicate (n = 3) and error bars denote SEM. (*E*) IMR-90 cells were treated with 0.5 pM truncated and bile-acid insensitive TcdB_1-2273_ (EC_99_) and increasing dilutions of the same sterile-filtered and boiled small intestinal, cecal, and colon contents as in *C*. Inhibition of TcdB_1-2273_-mediated cell rounding was calculated as TcdB-mediated cell rounding described above, experiments were conducted at n = 5 to 6, and error bars denote SD. (*F*) High-performance liquid chromatography (HPLC) elution method of sterile-filtered and boiled small intestinal content using acetonitrile:water and a C18 analytical column (Phenomenex). Fractions were collected every two min. (*G*) IMR-90 cells were treated with 0.5 pM TcdB (EC_99_) and fractions collected following HPLC fractionation of sterile-filtered and boiled small intestinal content. Experiment was conducted in singlicate (n = 1) due to fraction availability. Highlighted fraction, #7, was analyzed using untargeted tandem mass spectrometry (MS-MS). (*H*) Compounds enriched in fraction #7 and identified following untargeted MS-MS. Cholic acid, 7-ketodeoxycholic acid, and THCA were, respectively, enriched 394×, 129×, and 6× more in fraction #7 than in all the other fractions combined.

To determine whether and to what extent the extracted bile acids and other small molecules from intestinal contents and stool inhibit TcdB action on mammalian cells, processed extracts were serially diluted in SFM and added to human lung fibroblast (IMR-90) cells that were subsequently challenged with a cytopathic dose of TcdB that induces 99% cell rounding (i.e., 0.5 pM TcdB) (Fig. 1*A*). The sterile-filtered small-molecule contents extracted from both female and male mouse stool dose-dependently protected cells from TcdB-induced cell rounding to a similar degree (Fig. 1*B*). At the highest concentrations tested, corresponding to a 20-fold dilution in SFM, fecal extracts from female and male mice samples conferred 74% and 77% inhibition of TcdB-induced cell-rounding, respectively. Next, extracts of the lumenal contents from the small intestine, cecum, and colon of healthy mice were tested for their ability to inhibit TcdB intoxication using the same assay. In all cases, the extracted small molecules dose-dependently and completely inhibited TcdB-mediated cell rounding (Fig. 1*C*). Notably, the degree of protection and effective potency of the intestinal extracts was significantly greater than that of stool, suggesting that inhibitory molecules were in higher abundance in the intestine and, particularly, in the small intestine where complete protection from TcdB-induced cell rounding was observed at dilutions exceeding ~100-fold (Fig. 1*C*). The differential protection against TcdB observed, peaking with the contents extracted from the upper intestine followed by the lower intestine and then to stool, indicates that the abundance of the inhibitory molecules responsible for the protection in the small intestine exceeds that in the lower intestine and feces by approximately two orders of magnitude. This change in abundance closely mirrors the drop in total bile acid levels that results from the reabsorption of 95% of total bile acids in the distal ileum. This observation is consistent with bile acids being the major determinants of protection from TcdB. Below, we undertook additional experiments to support this.

### Evidence that Bile Acids in Small-Molecule Extracts Are Responsible for the Inhibition of TcdB.

To provide further evidence that bile acids are responsible for the inhibitory effects against TcdB seen with the intestinal extracts, we first tested whether the extracted contents were able to inhibit a variant of TcdB that lacks the bile acid binding site. Previously, we found that a C-terminally truncated form of TcdB lacking 83-residues from the C-terminus (TcdB_1-2273_)—though fully toxic to cells—lacked the bile acid binding site and was thus resistant to bile acid–mediated inhibition, while being sensitive to all other mechanisms of TcdB inhibition (21). Truncated TcdB_1-2273_ induces cell-rounding of human lung fibroblasts at similar doses as wild-type TcdB (Fig. 1*D*), and thus, we used the same fixed dose of TcdB_1-2273_ to test for inhibition by the intestinal extracts. At all concentrations tested, the extracted small-molecule contents from small intestine, cecum, and colon were unable to inhibit TcdB_1-2273_-mediated cell rounding (Fig. 1*E*), providing strong evidence that bile acids are primarily responsible for the inhibition seen by the intestinal extracts.

Next, to narrow down which bile acids within the extracts are responsible for inhibiting TcdB, we fractionated the small molecules from the small intestinal extract using a reverse-phase C18 analytical high-performance liquid chromatography (HPLC) column. A linear gradient of acetonitrile in water from 5 to 95% was run over 20 min, and ten fractions were collected in 2-min intervals (Fig. 1*F*). Each fraction was dried and resuspended in SFM before analysis by LC/MS and testing for activity against TcdB at its EC_99_. Among the ten fractions tested, only one major peak of inhibitory activity centered around fraction #7 was observed (Fig. 1*G*). To correlate activity to compound enrichment, each of the ten fractions were analyzed using untargeted LC/MS and compounds were identified using Thermo Scientific Compound Discoverer 3.2 and the following databases: The Human Metabolome Database, Kyoto Encyclopedia of Genes and Genomes, and BioCyc (Dataset S1). The level of each compound in an active fraction was calculated as a ratio of the area under the curve for the compound in that fraction to the sum of the areas under the curve for the compound in all of the other fractions (Dataset S2). Within fraction #7, only five compounds were significantly enriched relative to the inactive fractions, and mostly notably, three of these were bile acids: cholic acid (394-fold enriched); 7-ketodeoxycholic acid [7-kDCA] (129-fold enriched); and taurohyocholic acid [THCA] (sixfold enriched) (Fig. 1*H* and Dataset S3).

### Intestinal Extracts from Antibiotic-Treated and Germ-Free Mice Retain the Ability to Inhibit TcdB.

**Fig. 2.**
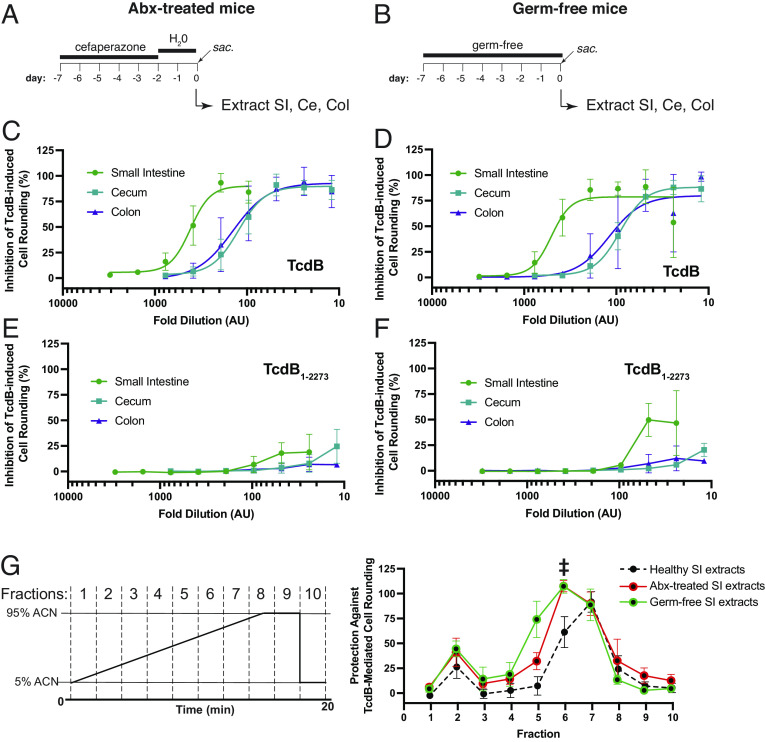
Intestinal bile acids from dysbiotic mice protect against *C. difficile* toxin B (TcdB). (*A*) C57BL/6 mice were treated with cefaperazone for 5 d and then stopped for 2 d before killing and collecting the luminal contents of the small intestine, cecum, and colon. (*B*) C57BL/6 germ-free mice were killed at a similar age as the antibiotic-treated C57BL/6 mice above. (*C*) Human lung fibroblast IMR-90 cells were treated with 0.5 pM TcdB (EC_99_) and increasing dilutions of sterile-filtered and boiled small intestinal, cecal, and colon content from antibiotic-treated mice, or from germ free mice (*D*). 0.5 pM truncated and bile acid–insensitive TcdB_1-2273_ (EC_99_) was tested against intestinal contents from antibiotic-treated mice (*E*) or germ-free mice (*F*). Inhibition of TcdB- or TcdB_1-2273_-mediated cell rounding was calculated as the ratio of the difference between the toxin- and gastrointestinal content-treated and healthy cells to toxin-treated and healthy cells, experiments were conducted at n = 6, and error bars denote (SD). (*G*) High-performance liquid chromatography (HPLC) elution method, top graph, of sterile-filtered and boiled small intestinal content using acetonitrile:water and a C18 analytical column (Phenomenex). Fractions were collected every 2 min. IMR-90 cells were treated with 0.5 pM TcdB (EC_99_) and fractions were collected following HPLC fractionation of sterile-filtered and boiled small intestinal contents of antibiotic-treated, germ free, or healthy mice, bottom graph. Experiments were conducted in triplicate (n = 3). Highlighted fraction, #6, from antibiotic-treated and germ-free mice small intestinal contents were analyzed using untargeted tandem mass spectrometry (MS-MS).

Having established that intestinal and fecal extracts from healthy mice protect against TcdB action and that bile acids are primarily responsible for this inhibition, we next asked whether the intestinal contents of antibiotic-treated and germ-free mice, both lacking an intact microbiota and thus harboring a distinctly different bile acid pool, were also able to inhibit TcdB. For the antibiotic-treatment group, C57BL/6 mice were treated with Cefoperazone from day 7 to day 2—a treatment paradigm that renders mice susceptible to CDI (22). At day 0, mice were killed and the small intestine, cecum, and colon lumenal contents were collected as before (Fig. 2*A*). In parallel, intestinal contents from germ-free mice, which are also susceptible to CDI, were extracted and processed as above (Fig. 2*B*). Extracted contents from antibiotic-treated (Abx-treated) and germ-free mice were serially diluted in SFM and tested against a cytopathic dose of TcdB. Unexpectedly, the extracted small molecules from the small intestine, cecum, and colon of both Abx-treated and germ-free mice fully and dose-dependently inhibited TcdB-mediated cell rounding (Fig. 2 *C* and *D*). In both cases, there was no significant inhibition of the truncated TcdB_1-2273_ construct (Fig. 2 *E* and *F*).

Given the dramatic change in the intestinal bile acid profile (and potentially other metabolites) in Abx-treated and germ-free mice relative to healthy mice, we next investigated which bile acids and/or other small-molecule metabolites in these extracts are responsible for the observed inhibition. Small intestinal extracts from Abx-treated and germ-free mice were fractionated using HPLC using the same protocol as was done above with healthy mice. Strikingly, the activity profile in fractionated samples from Abx-treated and germ-free mice were highly similar and both distinct from that seen for extracts from healthy mice. Although activity was centered around peak #7 with some activity in peak #6 for healthy mice (Fig. 2*G*), the patterns of activity for Abx-treated and germ-free mice extracts shifted and were more broadly centered around peak #6, with activities also seen in fractions #5 and #7 in both cases. For both Abx-treated mice and germ-free mice intestinal extracts, the most enriched compounds in the respective active fractions were bile acids; the taurine conjugated primary bile acid THCA was the most highly enriched compound in all active fractions from both Abx-treated mice and germ-free mice (Datasets S4–S7). Finally, to test whether any other non-bile acid-based small molecules might contribute to the protection afforded by the dysbiotic intestinal contents, we procured the most abundant nonbile acid small molecules that we identified previously in the intestinal content of mice following antibiotic treatment (23) and tested for their ability to inhibit TcdB-induced cell rounding. None of the 16 compounds, enriched in the intestinal of Abx-treated mice, tested showed any inhibitory activity of TcdB-mediated cell rounding (*SI Appendix*, Fig. S3).

### TcdB Levels and Total Bile Acid Levels Determine Sensitivity to Bile Acid-Mediated Inhibition.

**Fig. 3.**
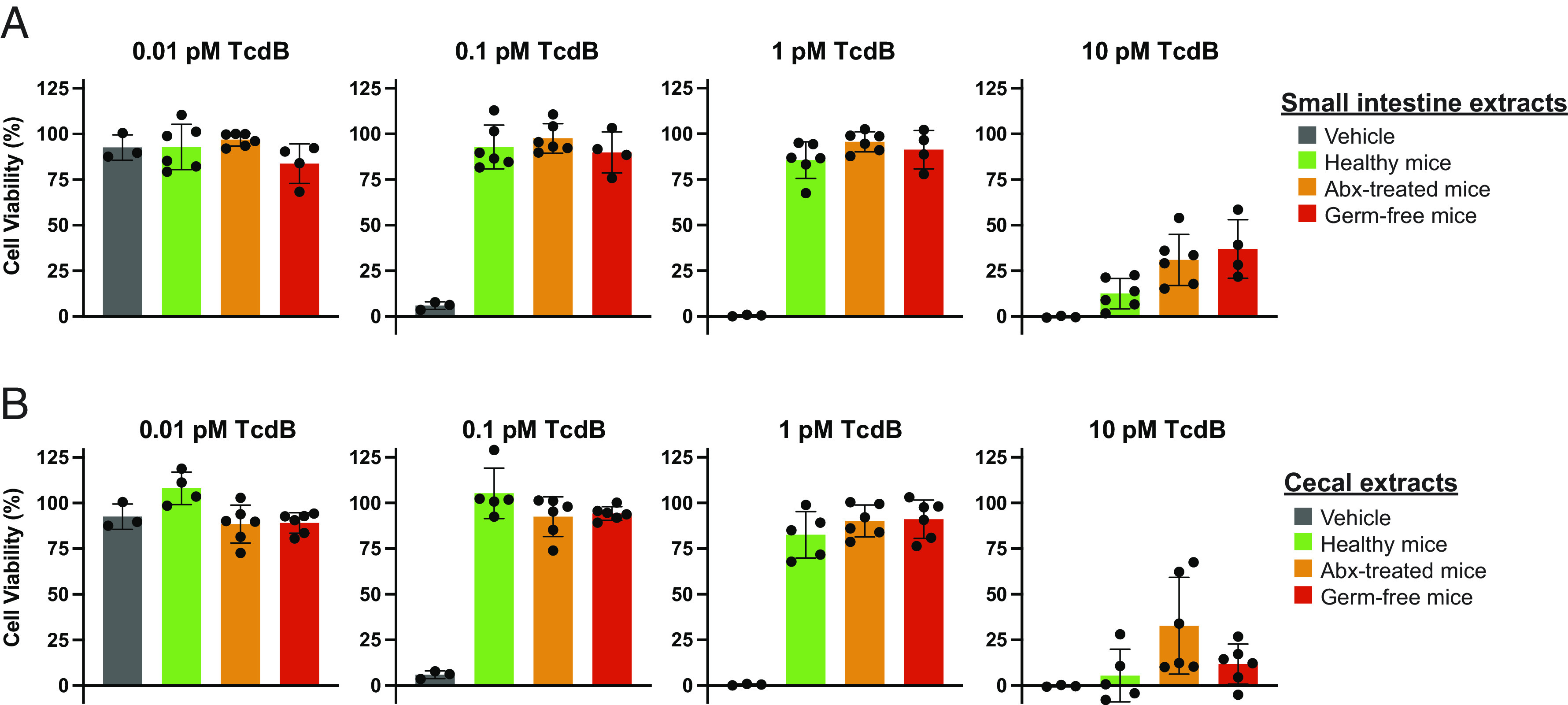
High *C. difficile* toxin B (TcdB) concentration overcomes bile acid–mediated protection afforded by the gastrointestinal content. Sterile-filtered and boiled small intestinal, (*A*) *Top* graphs, and cecal, (*B*) *Bottom* graphs, content of healthy, antibiotic-treated, and germ-free mice were tested against increasing concentration of TcdB. Intestinal content and vehicle control were incubated alone with nontoxigenic levels of TcdB (0.01 pM) on human lung fibroblast IMR-90 cells, left-most graphs. At 0.1 and 1 pM TcdB induces complete rounding of IMR-90 cells but is inhibited by small intestinal and cecal content of healthy, antibiotic-treated, and gem free mice. At 10 pM, TcdB also induces cell rounding but is well below the concentration at which it induces necrosis (>100 pM). High concentration of TcdB (≥10 pM) overcomes the bile acid–mediated protection afforded by healthy, antibiotic-treated, or germ-free mice small intestinal and cecal content, right-most graphs. Inhibition of TcdB-mediated cell rounding was calculated as the ratio of the difference between the toxin- and gastrointestinal content-treated and healthy cells to toxin-treated and healthy cells, experiments were conducted at n = 3 to 6, and error bars denote SD.

We have shown here that physiological levels of intestinal bile acids from healthy, Abx-treated, and germ-free mice protect against the cytopathic effects of TcdB, despite the fact that the latter two groups are ostensibly susceptible to TcdB action in vivo. To reconcile this apparent paradox, we next determined the extent of protection provided by the intestinal extracts against a wide range of concentrations of TcdB used to elicit cytotoxicity. For all of the challenge experiments performed above, intestinal extracts were titrated against a fixed dose of TcdB (viz., 0.5 pM) that causes ~99% of cells to round. However, TcdB concentrations in humans carrying TcdB-positive *C. difficile* have been reported to be as low as single-digit picomolar levels in individuals that are asymptomatic/mild symptoms to as high as two orders of magnitude more abundant in individuals with severe disease (*SI Appendix*, Table S1) (24, 25). We investigated whether different doses of TcdB, spanning this range, affected the degree of protection afforded by the intestinal extracts. In this paradigm, cells were treated with a range of concentrations of TcdB spanning four orders of magnitude in the absence and presence of intestinal extracts from the small intestine and cecum of healthy, Abx-treated, and germ-free mice (Fig. 3 *A* and *B*). Below the toxic threshold of TcdB (i.e., 0.01 pM), cells remained viable in the absence and presence of the small intestinal and cecal extracts, as expected. At the low and intermediate cytopathic doses of TcdB tested (i.e., 0.1 and 1 pM TcdB), small intestinal and cecal extracts offered complete protection from TcdB-induced effects. At 10 pM TcdB, however, the intestinal contents were no longer able to provide protection from TcdB-induced cytotoxicity, indicating that the baseline protection provided by bile acids is surmountable by higher TcdB levels.

Finally, we investigated whether supplementing the intestinal contents with a single bile acid could rescue cells that were treated with the highest dose of TcdB. Intestinal contents from healthy, antibiotic-treated, and germ-free mice were added to cells in the absence and presence of the conjugated secondary bile acid TLCA before being challenged by 10 pM TcdB. As before, the intestinal contents alone provided no protection against high-dose TcdB; however, in the presence of 400 μM TLCA, protection was restored (*SI Appendix*, Fig. S4). Taken together, these data highlight the delicate balance between TcdB and bile acids in modulating virulence and, importantly, demonstrate that increasing or supplementing the level of inhibitory bile acids in individuals with severe disease may be an effective strategy to reduce or prevent disease symptom onset.

### Total Bile Acid Levels in Human Stool Are Correlated With Protection against TcdB.

**Fig. 4.**
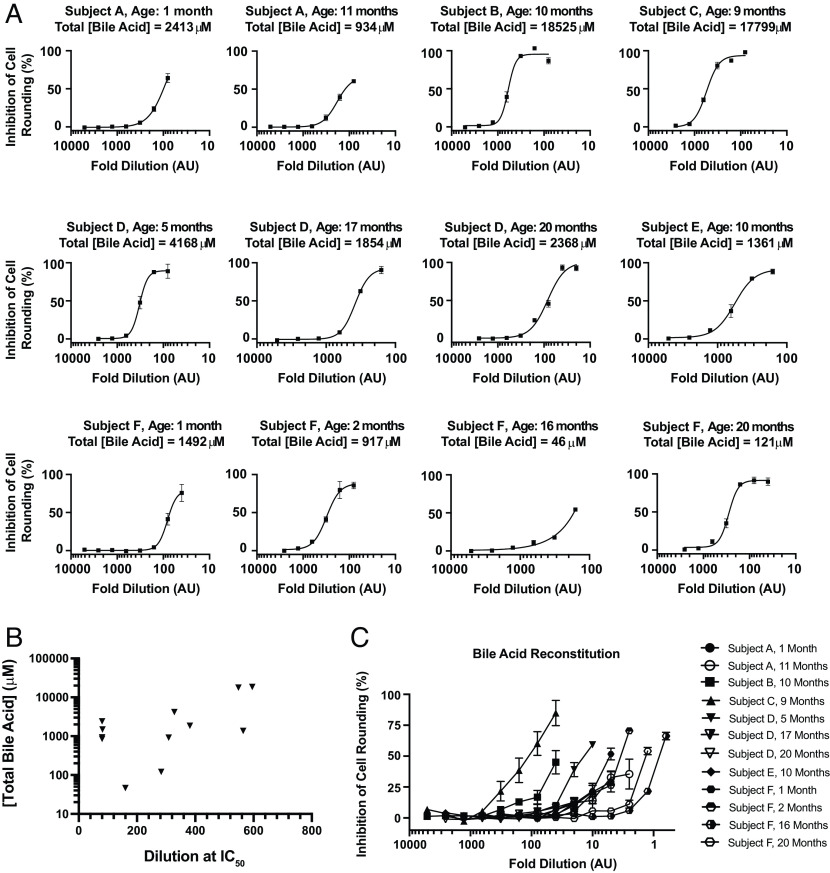
Bile acids from human stool protects against TcdB. (*A*) 12 stool samples from six different individuals (*A*–*F*) with ages indicated were collected and the small-molecule fraction was extracted. Samples were titrated against a fixed, cytopathic dose of TcdB (0.5 pM) and the degree of cell rounding was measured. (*B*) The total bile acids concentration for each of the twelve samples in A was determined using the Biocrates bile acid kid (see M&M) and plotted against the dilution required to achieve 50% inhibition of cell rounding. (*C*) Individual bile acid concentrations, as measured by the Biocrates Bile Acid mass spectrometry kit, were used to reconstitute the bile acid profile for each sample in SFM. Reconstituted samples were used to treat human lung fibroblast IMR-90 cells inoculated with a fixed dose of TcdB (0.5 pM). Experiment was conducted in triplicate (n = 3) and error bars denote (SEM).

Having demonstrated here that endogenous levels of intestinal and fecal bile acid extracts from murine-derived sources can protect against TcdB-induced effects, we next asked whether bile acids from human-derived samples were similarly protective against TcdB-induced effects. Twelve stool samples from six different healthy individuals under the age of two who were confirmed to be *C. difficile*-negative, and TcdB-negative were harvested and processed as above to extract the sterile small-molecule fraction. Samples titrated on human fibroblasts were challenged with a cytopathic dose of TcdB, and then, cell-rounding was quantified as above. Extracted contents from all 12 human stool samples dose-dependently protected against TcdB; however, the extent of protection seen at equivalent dilutions varied widely among the samples tested (Fig. 4*A*). For instance, whereas a 300-fold dilution of the extracted contents from subject B fully protected against TcdB-induced cell rounding, only partial protection was seen with the 16-fold diluted extracts from subject F at 16-mo. To elucidate the basis for the variability between samples, we measured the concentrations of individual bile acids and total bile acids in all samples and observed a similarly wide range in total bile acid concentration among the 12 samples (*SI Appendix*, Table S2). Comparing the total bile acid levels in each sample with its effective protection against TcdB-induced rounding revealed a strong correlation between the total bile acid concentrations measured in stool and the observed protection against TcdB (Fig. 4*B*).

Finally, to directly demonstrate that the specific bile acids derived from human stool are sufficient to protect against TcdB, we recreated the bile acid profile corresponding to each sample (*SI Appendix*, Table S2) and measured the ability of each mixture to protect against TcdB-induced cell rounding. Of note, because each mixture contained different amounts of each bile acid, which were dissolved from a stock DMSO solution, the highest doses that could be tested was limited by the final DMSO concentration that could be tolerated by human fibroblasts. The in vitro reconstituted mixtures, each containing a unique profile of bile acids corresponding to each of the samples, all demonstrated protection against TcdB-induced cell-rounding (Fig. 4*C*). As with the extracted samples assayed above (Fig. 4*A*), a wide range of protection was seen across samples. The results from the aforementioned two subjects exemplify this range, whereas for subject B (10 mo), more than 50% protection form cell rounding was seen at a 40-fold dilution; a similar level of protection for subject F (16 mo) required the sample to be twofold concentrated (Fig. 4*C*). Taken together, these data clearly demonstrate bile acids derived from mouse and human intestinal samples, despite large differences in their specific bile acid profiles (26), provide a barrier against *C. difficile* TcdB.

## Discussion

*C. difficile* is a major nosocomial pathogen that poses a substantial health and economic burden worldwide. Treating CDI with traditional antibacterial strategies (viz. antibiotics) is suboptimal owing to the unique features and complex lifecycle of the bacterium (27). Outside of the host, *C. difficile* exists primarily in its highly resilient spore form, which is resistant to antibiotics, biocides, and cleaning agents, thus increasing the chance of infection and reinfection, particularly in hospital settings (28). After being ingested, *C. difficile* spores exploit specific host factors within the gut to germinate into their vegetative form (1, 11, 12). In individuals whose microbiota is perturbed often as collateral damage from antibiotics for an unrelated infection, naturally resistant *C. difficile* is free to colonize the gut. Pathogenic strains of *C. difficile* produce up to three gut-damaging toxins (29, 30), including TcdB that is responsible for the gastrointestinal disease symptoms (29) which can range in severity from mild self-limiting diarrhea to life-threatening toxic megacolon (31–34). Encouragingly, however, the presence of *C. difficile* and TcdB do not necessarily portend disease as asymptomatic carriage of toxigenic *C. difficile* is common (35). Identifying the specific host and microbial factors that dictate the clinical outcome of CDI and understanding how they modulate disease pathogenesis offers hope for devising novel nonantibiotic approaches to attenuating or preventing CDI.

Bile acids have long been investigated for their role in modulating *C. difficile* lifecycle. Specific host-derived primary bile acids and microbiota-modified secondary bile acids have been shown to be key players in controlling both germination and vegetative growth of the bacterium (1, 11–16). Recently, we uncovered an unexpected additional role for bile acids in the *C. difficile* lifecycle, as direct inhibitors of TcdB pathogenesis in vitro (20, 36). We found that all of the primary and secondary bile acids tested inhibit TcdB-induced cell rounding, to varying extents. Bile acids were found to directly bind TcdB and induce a major confirmational change that prevents cell surface receptor engagement, the first step of the TcdB intoxication pathway. In the present study, we set out to explore the in vivo implications of the bile acid-TcdB axis by first investigating whether physiologically relevant levels of bile acids were capable of inhibiting TcdB in an ex vivo assay of toxin function. In mice, we found that small-molecule extracts from the intestinal lumen and feces of healthy mice dose-dependently inhibited TcdB-induced cell rounding in a bile acid–specific manner. This finding was consistent with our previous in vitro findings and supported the overall hypothesis that bile acids in healthy individuals contribute to the intrinsic resistance against *C. difficile*–induced disease. Moreover, despite dramatic differences in the intestinal bile acid profile between mice and humans (26), we found here that the inhibitory potential of the total intestinal bile acid pools in humans and mice is largely equivalent and capable of neutralizing low to moderate levels of TcdB. It is tempting to speculate, based on these data, that bile acids might be responsible for neutralizing TcdB in asymptomatic carriers of toxigenic *C. difficile* and preventing symptom onset. Our findings that bile acids extracted from antibiotic-treated and germ-free mice also offered protection against TcdB-induced cell rounding, however, suggested a more nuanced role of bile acids in *C. difficile* disease pathogenesis. A key insight into the interplay between bile acids and TcdB came from our demonstration that protection by bile acids could be overcome with higher levels of TcdB—such as those seen during more severe disease, which is correlated with higher toxin levels (24, 25).

**Fig. 5.**
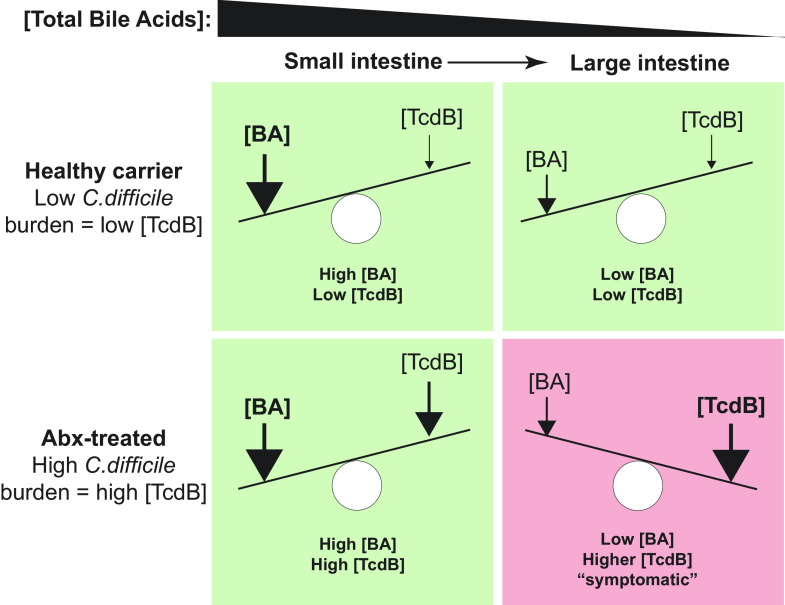
Model of bile acid mediated protection in asymptomatic carriers and the transition to symptomatic disease at high *C. difficile* toxin B (TcdB) levels. Bile acid concentration decreases as the gastrointestinal tract transition from small intestine to large intestine due to the active reabsorption of bile acids in the terminal ileum. In the small intestine, the high bile acid concentration protects against the low levels of TcdB. In a dysbiostic gut, following antibiotic treatment, the altered bile acid pool provides a conducive environment of *C. difficile* growth and toxin production yet is still sufficient to protect against the tolerable levels of TcdB. No TcdB-mediated damage or symptoms can be experienced in the small intestine of healthy or antibiotic-treated (Abx-treated) organisms. In the large intestine of healthy carriers, the bile acid concentration decreases but is still sufficient to protect against the low levels of TcdB. In the large intestine of antibiotic-treated organisms, the lack of secondary bile acids and concomitant increase in primary bile acids further promotes *C. difficile* proliferation and toxin production. The significant increase in TcdB levels overcomes the protection afforded by bile acids here and leads to TcdB-mediated damage and disease symptoms.

Taken together, we propose a model here for the role of bile acids in modulating TcdB that accounts for the dynamic interplay between the changing levels of total bile acids within different regions of the intestine and between individuals, the total *C. difficile* burden, and the vast differences in TcdB concentration in individuals (Fig. 5). This model posits that TcdB-mediated damage would only occur at high levels of TcdB and only in the lower intestine, where *C. difficile* normally proliferates and secretes toxin (37) and where bile acids have dropped below the inhibitory threshold due to enterohepatic reabsorption of bile acids. An important tenet of this model is that the higher levels of total bile acids in the upper intestine are sufficiently high as to protect regardless of TcdB levels—consistent with toxin-mediated disease pathology being confined to the lower intestine (37). This model provides a conceptual framework for understanding how the dynamic balance between toxicity and protection can be tilted toward the latter by increasing the inhibitory capacity of the intestinal bile acid pool through supplementation. Any such approaches will, however, need to carefully consider the consequences of bile acid supplementation on the host and microbiota (38).

Another potential implication of this work is that bile acids that are transplanted along with the donor stool during fecal microbiota transplantation (FMT) may contribute to its remarkable efficacy in treating CDI recurrence. In support of this, Ott *et al*. recently showed that sterile fecal filtrate showed efficacy in treating CDI (39). A preliminary study with five patients experiencing CDI recurrence showed that transfer of sterile filtrates from donor stool, rather than fecal microbiota, was sufficient to restore normal stool habits and eliminate symptoms. Additional studies with more patients are required to verify this important finding that could usher in a new era of microbe-free FMT. Microbe-free FMT could potentially maintain the excellent clinical effectiveness of FMT while greatly reducing risk of infection-related complications in recipients, particularly those of epidemiological importance such as transmission of multidrug-resistant organisms and/or emerging pathogens.

In conclusion, the work presented here extends the interaction between bile acids and TcdB in vitro (20) and highlights the dynamic interplay between bile acid and TcdB within the gut, the levels of which may ultimately determine whether toxin-dependent damage to the host occurs.

## Materials and Methods

### Mouse Fecal Samples Preparation.

Fecal samples were collected from two cages containing three male C57BL/6 TPP1 knockout mice each and one cage containing four female C57BL/6 TPP1 knockout mice. Fecal samples were weighted and resuspended in serum-free EMEM (Multicell) supplemented with 1% of penicillin/streptomycin (SFM) at 1 μL per 1 μg of sample using a vortex mixer and bath sonicator for 1 min. Samples were centrifuged at max speed for 10 min at 4 °C. The supernatant was then sterile filtered using a 0.22-μm spin filter (Corning Costar). The filtrate was heat denatured at 90 °C for 10 min and then mixed using a vortex mixer for 10 s, followed by centrifugation at max speed for 10 min at 4 °C. The sterile-filtered and heat-denatured supernatant was used for subsequent experiments.

### Mouse Fecal Sample Preparation Tested against TcdB.

Sterile-filtered and heat-denatured mouse fecal samples were tested for inhibition of TcdB-mediated cell rounding. Human lung fibroblast IMR-90 cells American Type Culture Collection (ATCC) were seeded in 96-well clear CellBind plates (Sigma Aldrich) at least 24 h prior to the experiment at a density of 3,000 cells/well in a 100 μL volume of complete EMEM (Multicell) supplemented with 10% inactive fetal bovine serum (FBS) and 1% of penicillin/streptomycin. On the day of the experiment, the media was replaced with 100 μL of SFM containing 1 μM CellTracker™ Orange CMRA Dye (ThermoFisher), and cells were incubated at 37 °C for at least 1 h. Sterile-filtered and heat-denatured mouse fecal samples were serially diluted twofold using SFM for up to eight dilutions. SFM media containing 1 μM CellTracker™ Orange CMRA Dye (ThermoFisher) was aspirated. The dilution series was then added to IMR-90 cells (ATCC) followed by the addition of 0.5 pM TcdB and incubated at 37 °C for 3.5 h. Cells were imaged with a Cellomics ArrayScan (Thermo Scientific). TcdB-mediated toxicity was assessed using the cell rounding index, as described before (40). The product of the cell’s total area and length-to-width ratio produced a cell rounding index. Sterile-filtered and heat-denatured fecal sample treated well’s total inhibition was calculated using the formula: $\begin{array}{c} Inhibition\left(\%\right)=\frac{treatedcells-fullyroundedcells}{healthycells-fullyroundedcells}\times 100\% \end{array}$ . Data were plotted using Prism 9 (GraphPad).

### Animals and Housing at North Carolina State University.

Male and female C57BL/6J mice (aged 5 wk old) were purchased from Jackson Labs (Bar Harbor, ME) for use in infection experiments. The food, bedding, and water were autoclaved, and all cage changes were performed in a laminar flow hood. The mice were subjected to a 12-h light and 12-h dark cycle. Animal experiments were conducted in the Laboratory Animal Facilities located on the NCSU CVM campus. The animal facilities are equipped with a full-time animal care staff coordinated by the Laboratory Animal Resources division at NCSU. The NCSU CVM is accredited by the Association for the Assessment and Accreditation of Laboratory Animal Care International. Trained animal handlers in the facility fed and assessed the status of animals several times per day. Those assessed as moribund were humanely euthanized by CO2 asphyxiation. This protocol is approved by NC State’s Institutional Animal Care and Use Committee.

### Mouse Intestinal Tract Samples Preparation.

Healthy C57BL/6 mice were killed and gastrointestinal tract content from the small intestine, cecum, and colon were collected and separately stored at −80 °C. Antibiotic-treated C57BL/6 mice were treated with cefaperazone for 5 d, followed by no treatment for 2 d. Afterward, mice were killed and small intestinal, cecal, and colon contents were collected as above. Germ-free C57BL/6 mice were killed, and small intestinal, cecal, and colon contents were collected as above. All gastrointestinal tract samples were prepared using the same protocol as the mouse fecal samples above.

### Mouse Intestinal Tract Sample Tested against TcdB.

Sterile-filtered and heat-denatured mouse gastrointestinal tract samples were tested for inhibition of TcdB-mediated cell rounding using the same protocol as above. Data were plotted using Prism 9 (GraphPad). Additionally, sterile-filtered and heat-denatured mouse gastrointestinal tract samples were tested for inhibition of the bile-acid–insensitive TcdB_2273_-mediated cell rounding (21) using the same protocol as above but with the use of 0.5 pM TcdB_2273_ instead of full-length TcdB.

Sterile-filtered and heat-denatured mouse gastrointestinal tract samples were also tested against increasing concentration of toxins. For this experiment, 96× diluted small intestinal and 24× diluted cecal and colon sterile-filtered and heat-denatured samples were used. Then, 100 pM TcdB or 100 pM TcdB_2273_ were serially diluted threefold by adding two parts SFM for one part toxin solution for up to ten dilutions. Following the above protocol and after the aspiration of SFM media containing 1 μM CellTracker™ Orange CMRA Dye (ThermoFisher), the sterile-filtered and heat-denatured mouse gastrointestinal tract samples were added to IMR-90 cells (ATCC) followed by the addition of increasing concentration of TcdB or TcdB_2273_ and incubated at 37 °C for 3.5 h. Toxin-only controls were also used, where 2 pM TcdB or 2 pM TcdB_2273_ were serially diluted threefold by adding two parts SFM for one part toxin solution for up to eight dilutions. Following the aspiration of SFM media containing 1 μM CellTracker™ Orange CMRA Dye (ThermoFisher), SFM was added to IMR-90 cells (ATCC) followed by the addition of increasing concentration of TcdB or TcdB_2273_ and incubate at 37 °C for 3.5 h. Cells were imaged and processed as before. Data were plotted using Prism 9 (GraphPad).

Small intestinal tract contents of healthy and antibiotic-treated mice were processed as above and diluted 96-fold. The samples were exogenously supplemented with TLCA at 400 μM. Inhibition of TcdB-mediated cell rounding was tested as above. Data were plotted using Prism 9 (GraphPad).

### Mouse Fecal Samples Incubation on LB Agar.

Mouse fecal samples were prepared using the same protocol as above. After each step of the preparation process, a small volume was set aside. All of these samples were then spread on LB agar plates and incubated at 37 °C overnight. Images were taken of the top and bottom of the LB agar plate.

### Healthy Mouse Intestinal Tract Samples Tested for Compound-Mediated Toxicity before and after Boiling.

Healthy C57BL/6 mouse small intestinal, cecal, and colon samples were prepared using the same protocol above. Before boiling, half of each sample was set aside. Both the boiled and nonboiled small intestinal, cecal, and colon samples were serially diluted twofold using SFM for up to eight dilutions. To test for compound-mediated toxicity, IMR-90 cells (ATCC) were prepared as above. Once ready, the media were replaced with 100 μL of serially diluted small intestinal, cecal, and colon samples, and cells were incubated at 37 °C for 3.5 h. Afterward, 10 μL of PrestoBlue (ThermoFisher) was added to 100 μL of media containing cells. After a 2-h incubation, fluorescence was measured using a Spectromax m5e (Molecular Devices) at an excitation of 555 nm and emission of 585 nm. Data were plotted using Prism 9 (GraphPad).

### Bile Acid Sample Extraction Analysis Using Mass Spectrometry.

All ten bile acids were spiked together into SFM at a final concentration of 100 μM (TLCA at 10 μM). The bile acid-spiked SFM was manipulated using the protocol used to prepare the fecal and gastrointestinal tract contents of mice. Bile acids in spiked SFM were identified using a Thermo Scientific Q Exactive mass spectrometer (Thermo Scientific) with an HESI II source and at a spray voltage of 3.5 kV with an upstream Thermo Scientific Ultimate 3,000 (Thermo Scientific) ultrahigh performance liquid chromatography (UHPLC) with a BEH C18 analytical column (Waters) at a column temperature of 50 °C. Samples were fractioned using increasing concentration of acetonitrile: 20 mM ammonium acetate and 20 mM ammonium hydroxide in water at a flow rate of 0.3 mL/min. The gradient used was as follows: 0 to 1 min, 10% acetonitrile; 1 to 5 min, linear gradient to 100% acetonitrile; 5 to 10 min, 100% acetonitrile; 10 to 10.5 min, linear gradient to 10% acetonitrile; 10.5 to 15 min, 10% acetonitrile. Samples were detected at a mass-to-charge (m/z) range of 100 to 1,000 in both positive and negative modes. Bile acid–targeted data processing was done using Thermo Scientific Xcalibur Qual Browser (Thermo Scientific) with an error window of five parts per million (ppm) from the monoisotopic parent molecule mass. The area under the curve of each bile acid at each step of the preparation process was graphed using Prism 9 (GraphPad).

Small intestinal tract content of healthy, antibiotic-treated, and germ-free mice at 24-fold dilution was fractioned using reverse-phase HPLC (Waters) and an analytical biphenyl column (Phenomenex). The column, set at 35 °C, was equilibrated at 95% water with 0.1% formic acid. Elution of the samples was done using acetonitrile at a flow rate of 1 mL/min and the following method: Start at 5% acetonitrile with 0.1% formic acid and increase concentration of acetonitrile linearly to 95% for 15 min, hold at 95% for 3 min, then switch to 5%, and hold for 2 min for a total run time of 20 min. Fractions were individually collected in 2-min intervals and dried using a centrifugal evaporator (Genevac). Fractions were resuspended using SFM matching the volume to what was used to initially fraction the samples using HPLC. Fractions were then tested for inhibition of TcdB-mediated cell rounding using the same protocol as above. Each fraction was analyzed using mass spectrometry as described above for the bile acid–spiked SFM. In this case, untargeted data processing was done using Thermo Scientific Compound Discoverer 3.2 (Thermo Scientific) and the following databases: The Human Metabolome Database, Kyoto Encyclopedia of Genes and Genomes, and BioCyc. The most enriched compounds were identified by comparing the area under the curve of each identified compound in the active fractions to the sum of the areas under the curve of the compound in all of the inactive fractions.

### Bile Acid Half-Maximal Effective Concentration (EC_50_) Analysis.

Bile acids were tested for inhibition of TcdB-mediated cell rounding using the same protocol as above with two modifications: 1) Following the aspiration of SFM media containing 1 μM CellTracker™ Orange CMRA Dye (ThermoFisher), bile acid-containing SFM was then added followed by the addition of 0.5 pM TcdB, and 2) a duplicate solution of bile acid-containing SFM was heat denatured at 90 °C for 10 min and then mixed using a vortex mixer for 10 s, followed by centrifugation at max speed for 10 min at 4 °C before addition to IMR-90 cells (ATCC). Data were plotted using Prism 9 (GraphPad).

### Identification of Compounds Enriched in the Intestinal Tract of Mice Following Antibiotic Treatment.

List of metabolites identified in the gastrointestinal tract of healthy and antibiotic-treated C57BL/6 mice from Theriot et al. (23) was extracted. Metabolites with a positive fold change following antibiotic treatment were enriched in antibiotic-treated mice gastrointestinal tract compared to healthy mice. These metabolites were purchased (Sigma) in powdered form, resuspended in dimethyl sulfoxide, and tested for inhibition of TcdB-mediated cell rounding using the same protocol as above.

### Infant Fecal Content.

Stool samples for the present study were selected from a biobank of samples previously collected from infants through an unrelated prospective cohort study (41) performed at the Ann & Robert H. Lurie Children’s Hospital of Chicago and approved by their Institutional Review Board. Healthy full-term infants ≤ 2 m old without previous admission to a neonatal intensive care unit were enrolled through the general pediatrics clinic between September 2014 and January 2017. Parents were requested to provide infant stool samples at each well-child visit between enrollment and 2 y of age. Stools from infants were derived from soiled diapers provided by the parents. Stool was extracted from diapers, aliquoted, and stored at −80 °C until ready for batch processing of study-related assays. Thawed infant stool samples underwent several assays to identify toxigenic and nontoxigenic *C. difficile*, including glutamate dehydrogenase and toxin A/B enzyme immunoassay, as well as tcdB (toxin B gene) PCR testing and anaerobic stool culture. Infant stools included in this study were negative for toxigenic and nontoxigenic *C. difficile* using all of these assays.

Fecal samples were lyophilized to remove excess water content. Reweighed samples were resuspended in SFM at 1 μL per 1 μg of dry sample weight using a vortex mixer and bath sonicator for 1 min. The samples were then prepared using the same protocol as the mouse fecal samples above.

### Infant Fecal Content Tested against TcdB.

Sterile-filtered and heat-denatured infant fecal samples were tested for inhibition of TcdB-mediated cell rounding using the same protocol as above. Data were plotted using Prism 9 (GraphPad).

### Infant Fecal Content Bile Acid Levels.

Sterile-filtered and heat-denatured infant fecal samples endogenous bile acid levels were quantified using the Biocrates’ Bile Acids Kit with the help of the Analytical Facility for Bioactive Molecules at The Hospital for Sick Children in Toronto, Canada.

## Supplementary Material

Appendix 01 (PDF)

Dataset S01 (XLSX)

Dataset S02 (XLSX)

Dataset S03 (XLSX)

Dataset S04 (XLSX)

Dataset S05 (XLSX)

Dataset S06 (XLSX)

Dataset S07 (XLSX)

## Data Availability

All study data are included in the article and/or supporting information.
