# Supplementary material for: Intestinal bile acids provide a surmountable barrier against C. difficile TcdB-induced disease pathogenesis
Source: Proc Natl Acad Sci U S A. 2023 May 1;120(19):e2301252120. doi: 10.1073/pnas.2301252120 (PMC10175849; doi:10.1073/pnas.2301252120)
Supplement: Supplementary file 1 — Appendix 01 (PDF) [file pnas.2301252120.sapp.pdf]

Intestinal bile acids provide a surmountable barrier against *C. difficile* TcdB-induced disease pathogenesis

Simoun Icho[1,2], Jennifer S. Ward[1,2], John Tam[1], Larry K. Kociolek[3], Casey M. Theriot[4], Roman A. Melnyk[1,2,*]

Supplemental Figures

A

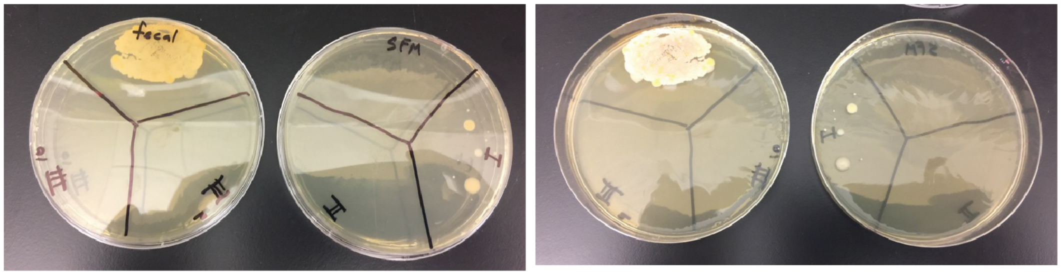

SFM – serum free media
Fecal – mouse C57BL/6 fecal samples
I – centrifuged
II – sterile filtered
III5 – 5 min boiling
III10 – 10 min boiling

B

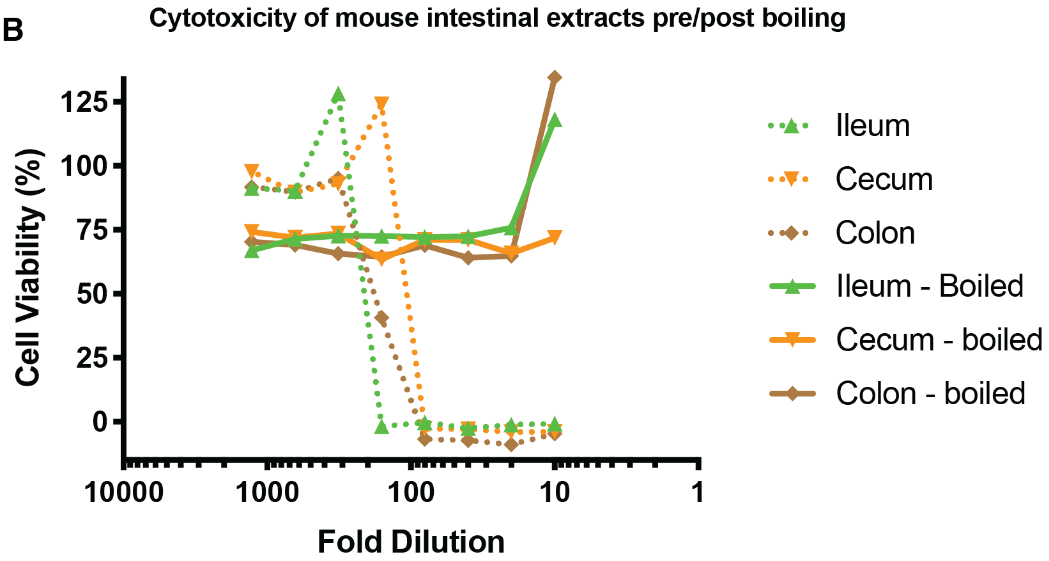

Supplemental Figure 1. Sterility and toxicity of fecal and intestinal content of healthy C57BL/6 mice. (A) Fecal content of healthy C57BL/6 mice were extracted using the protocol from Figure 3.1. Samples from each step of the extraction protocol were grown on Luria-Bertani agar plates overnight at 37°C. The boiling step was tested at both five and ten minutes. (B) Sterile filtered ileal, cecal, and colon samples of healthy C57BL/6 mice before and after boiling for ten minutes were tested for compound-mediated toxicity against human IMR-90 lung fibroblast cells. Experiment was done in singlicate (n=1).

A

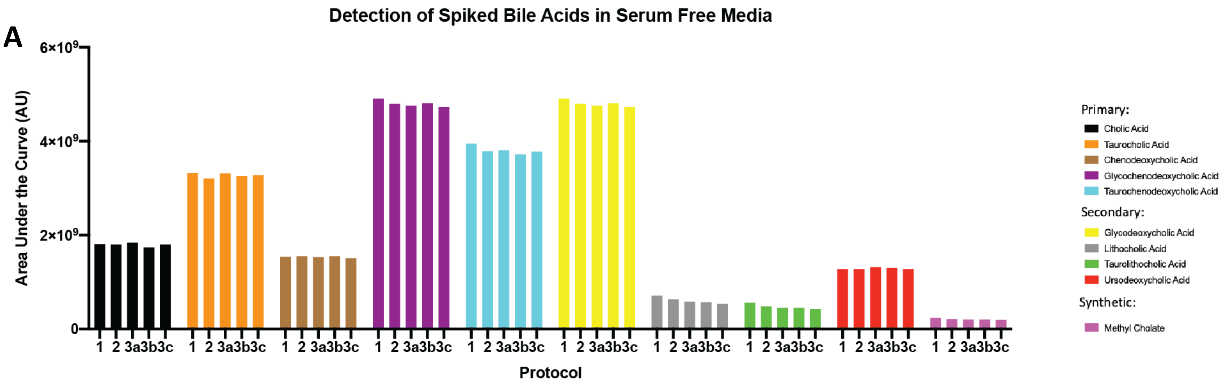

1 - Starting material in SFM
2 - Sterile filtered supernatant of (1)
3 - Supernatant from (1) boiled for 2 minutes
4 - Supernatant from (1) boiled for 5 minutes
5 - Supernatant from (1) boiled for 10 minutes

B

Inhibition of Cell Rounding (IC50 in μM)	Before extraction protocol	After extraction protocol
**Primary:**		
Cholic Acid	258	223
Chenodeoxycholic Acid	59	71
Glycochenodeoxycholic Acid	141	147
Taurochenodeoxycholic Acid	78	80
**Secondary:**		
Glycodeoxycholic Acid	120	254
Taurolithocholic Acid	19	14
**Synthetic:**		
Methyl Cholate	7	14

Supplemental Figure 2. Extraction protocol neither alters bile acid abundance or inhibition of *Clostridioides difficile* toxin B (TcdB). (A) Five primary, four secondary, and one synthetic bile acids were spiked into serum free media and then measured for using liquid chromatography mass spectrometry following each step of the extraction protocol. (B) Four primary, two secondary, and one synthetic bile acid effective against TcdB were boiled for ten minutes and retested for inhibitory activity against TcdB. half-maximal inhibitory concentrations were extracted from experiments conducted in triplicate (n=3).

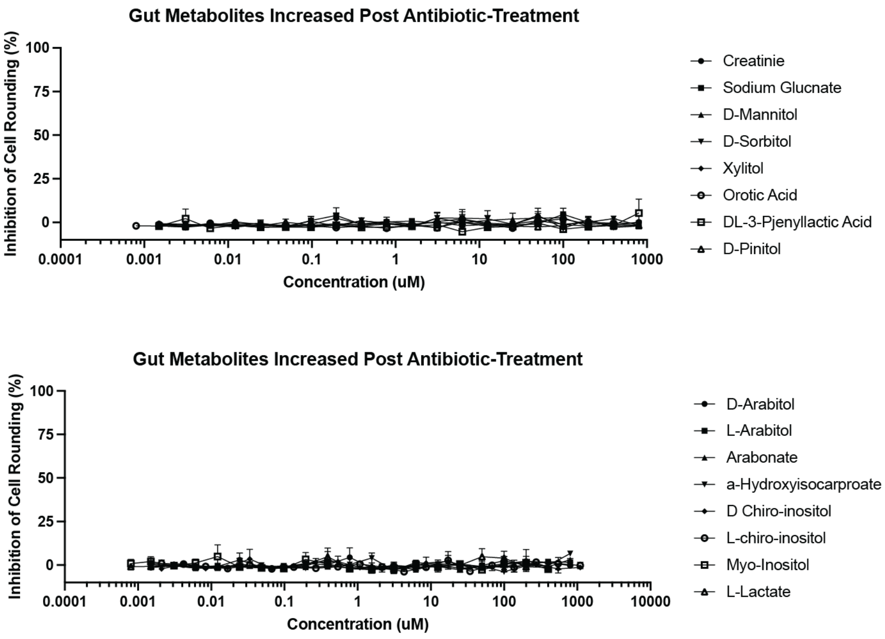

Supplemental Figure 3. Non-bile acid compounds enriched in the intestinal content of antibiotic-treated C57BL/6 mice are inactive against *Clostridioides difficile* toxin B (TcdB). Sixteen non-bile acid compounds enriched and identified in the intestinal content of C57BL/6 mice following cefaperazone treatment were tested against TcdB on IMR-90 cells at concentrations up to 1mM. Experiments were done in triplicate (n=3) and error bars denote standard error of the mean.

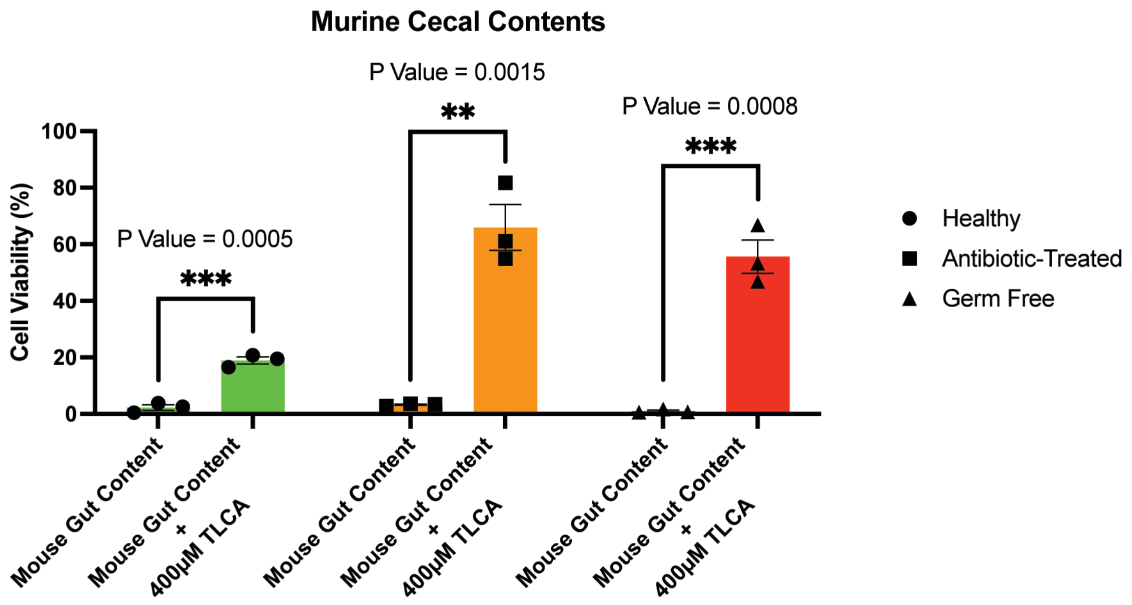

Supplemental Figure 4. Supplementing TLCA to intestinal extracts that are not protective against high concentrations of TcdB restores protection. Extracts from healthy, antibiotic-treated and germ-free mice were unable to protect against cytotoxicity to human IMR-90 cells induced by 10pM of TcdB. Addition of 400uM TLCA to these extracts, significantly restored the protection against TcdB induced cytotoxicity.

Disease Severity	[TcdB] from Ryder *et al.* 2010	[TcdB] from Huang *et al.* 2014
No Clinical Symptoms	1 pM	8 pM
Mild	5 pM	11 pM
Mild to Moderate	124 pM	18 pM
Moderate	58 pM	112 pM
Moderate to Severe	215 pM	191 pM
Severe	413 pM	Not Available

Supplemental Table 1. Concentration of *Clostridioides difficile* toxin B (TcdB) in the fecal content of patients with varying disease severity.

		CA	GCA	TCA	CDCA	GCDCA	TCDCA	LCA	DCA	HDCA
	Binding EC50 (uM)	190	340	530	70	160	120	42	110	279
	Concentration (uM)									
Patient #	Month	CA	GCA	TCA	CDCA	GCDCA	TCDCA	LCA	DCA	HDCA
4	1	1368.00	0.00	0.00	124.00	0.00	0.00	0.00	0.00	0.00
4	2.2	780.80	0.00	0.00	103.20	0.00	0.00	0.00	0.00	32.80
4	4.1	304.80	0.00	0.00	20.00	0.00	0.00	0.00	0.00	12.80
4	6.1	12.00	0.00	0.00	12.80	0.00	0.00	6.40	302.40	2.40
4	9.7	64.80	0.00	0.00	61.60	0.00	0.00	3.20	583.20	14.40
4	12.2	4.80	0.00	2.40	2.40	0.00	0.00	10.40	728.80	3.20
4	15.7	9.60	0.00	0.00	1.60	0.00	0.00	8.00	24.00	3.20
4	19.9	7.20	0.00	0.00	0.80	0.00	0.00	4.80	105.60	2.40
7	1.8	4.00	0.00	0.00	0.80	0.00	0.00	4.80	168.00	1.60
7	5.2	3032.00	4.80	11.20	727.20	0.00	0.00	0.00	372.00	20.80
7	16.9	21.60	0.00	0.00	13.60	0.00	0.00	60.00	1752.00	6.40
7	19.6	35.20	0.00	0.00	41.60	0.00	0.00	67.20	2208.00	16.00
11	2.9	2016.00	0.00	0.80	214.40	0.00	0.00	0.00	0.00	1.60
11	4.6	2200.00	8.00	2.40	161.60	0.00	0.00	0.00	0.00	96.00
11	6.7	771.20	0.00	0.00	231.20	0.00	0.00	0.00	0.00	23.20
11	10.1	60.00	0.00	0.00	26.40	0.00	0.00	30.40	1232.00	12.00
19	4.4	204.80	0.00	0.00	7.20	0.00	0.00	0.80	0.00	8.80
19	5.8	261.60	0.00	0.00	31.20	0.00	0.00	0.00	0.00	33.60
19	7.2	1216.00	0.80	0.00	82.40	0.00	0.00	0.00	0.00	51.20
19	10.2	5.60	0.00	0.00	0.00	0.00	0.00	12.00	604.80	2.40
28	1.1	415.20	0.00	8.00	70.40	0.00	0.00	0.00	9.60	1.60
28	6.2	2064.00	0.00	0.00	886.40	0.00	0.00	0.00	0.00	3.20
28	9.2	11840.00	5.60	8.80	5920.00	4.80	4.00	0.00	0.00	16.00
29	4.4	2792.00	23.20	75.20	1152.00	1.60	0.00	0.80	0.00	67.20
29	6.1	1392.00	0.00	0.00	200.00	0.00	0.00	0.00	0.00	17.60
29	10.1	2704.00	11.20	0.00	176.00	0.00	0.00	0.00	0.00	56.00
31	2	341.60	0.00	0.00	67.20	0.00	0.00	0.80	1.60	1.60
31	4.6	33.60	0.00	0.00	15.20	0.00	0.00	0.00	184.80	4.00
31	5.5	5144.00	18.40	5.60	1520.00	3.20	0.00	1.60	464.80	73.60
31	10.2	15200.00	36.80	16.80	2792.00	2.40	0.00	0.00	318.40	158.40
34	1	2016.00	10.40	0.80	383.20	0.00	0.00	0.00	0.00	2.40
34	3.1	6.40	170.40	88.80	1.60	164.80	48.80	0.00	0.00	0.00
34	6.4	1337.60	4.80	0.00	529.60	0.00	0.00	0.00	0.00	3.20
34	11.3	775.20	0.00	0.80	119.20	0.00	0.00	0.00	0.00	38.40
41	1.9	94.40	0.00	0.00	9.60	0.00	0.00	0.00	0.00	6.40
41	4.1	1904.00	6.40	78.40	99.20	0.00	0.00	0.00	0.00	20.80
41	6.4	132.80	0.00	10.40	153.60	2.40	8.80	0.00	0.00	24.00
41	9.2	156.80	0.00	0.00	4.80	0.00	0.00	0.00	0.00	11.20

Supplemental Table 2: Human Fecal Bile acid levels. Sterile filtered and heat denatured infant fecal samples endogenous bile acid levels were quantified using the Biocrates' Bile Acids Kit
